# Beyond broken spines–what the radiologist needs to know about late complications of spinal cord injury

**DOI:** 10.1007/s13244-014-0375-8

**Published:** 2014-12-11

**Authors:** Erin Capps, Ken F. Linnau, Deborah A. Crane

**Affiliations:** 1 Department of Radiology, The Queen’s Medical Center, 1301 Punchbowl Street, Honolulu, HI 96813 USA; 2 Department of Radiology, University of Washington/Harborview Medical Center, 325 9th Avenue, Box 359728, Seattle, WA 98104 USA; 3 Department of Rehabilitation Medicine, University of Washington/Harborview Medical Center, 325 9th Avenue, Box 359740, Seattle, WA 98104 USA

**Keywords:** Spinal cord injury, Complications, Long term effect, Computed tomography, Multidetector radiography

## Abstract

**Objective:**

To describe expected imaging findings to assist the emergency room radiologist with recognising complications and pathology unique to the spinal cord injury (SCI) patient population to ensure rapid and accurate diagnosis.

**Methods:**

Pictorial review.

**Results:**

We review several imaging findings common to persons with chronic SCI, emphasising imaging in the emergency setting and on CT.

**Conclusion:**

SCI patients present a unique diagnostic challenge, as they may present with symptoms that are difficult to localise because of abnormal sensation and autonomic instability. Imaging plays an important role in the emergent setting, rapidly differentiating the most commonly encountered complications from less common, unanticipated complications. Radiologists need to be attuned to both the expected findings and potential complications, which may be unique to SCI patients, to ensure accurate diagnosis and treatment in the emergency setting.

**Main Messages:**

• Medical complications after spinal cord injury are common and associated with significant morbidity.

• Radiologists should be aware of complications unique to the SCI population to aid diagnosis.

• Due to abnormal sensation, SCI patients often present with symptoms that are difficult to localise.

• In the ED, imaging helps to rapidly differentiate common complications from less anticipated ones.

Spinal cord injury (SCI) is not uncommon. In the USA, it is estimated that there are 12,000 new cases of traumatic SCI each year and about 265,000 persons living with traumatic SCI at present [1]. Significant improvements have been made in the management of spinal cord injury. For individuals surviving beyond 1 year post SCI, life expectancy is estimated at 90 % of that of the general population [2, 3]. Despite these significant improvements in survival and life expectancy, medical complications after SCI are common and associated with significant morbidity. Spinal cord injured patients are frequently evaluated and imaged in the emergency room setting. To ensure rapid and accurate diagnosis, it is essential that the radiologist be familiar with expected imaging findings, recognise complications and pathology unique to the SCI patient population, and be knowledgeable of emergency conditions and potential pitfalls in diagnosing and treating SCI patients.

Spinal cord injury produces a wide variety of changes in the systemic physiology, which can lead to numerous complications. Re-hospitalisation occurs in 55 % of patients in the first year following injury and continues at a steady rate of about 37 % per year over the next 20 years [4]. Urinary and skin complications account for the most common indications for hospital re-admission [5].

We will review the imaging findings common to persons with chronic SCI, emphasising imaging in the emergency setting and on CT. We have also included a table including practical ‘pearls and pitfalls’ for readers’ benefit (Table 1).

**Table 1:** Pearls and pitfalls

Organ system	Pearls	Pitfalls
GU	Pseudohydronephrosis defined as a focal enlargement of the urinary tract can usually be distinguished from urinary obstruction on CT because it lacks asymmetric fat stranding, unilateral ureter dilatation and unilateral renal enlargement*	Self-catheterisation or urological instrumentation can introduce gas in the collecting system and carful clinical history allows differentiation from pyelitis
GI		Obtain abdominal imaging early for non-specific symptoms such as nausea, anorexia or autonomic dysreflexia to avoid delayed diagnosis of cholecystitis and pancreatitis
Cardiovascular	CAD and PE imaging results in SCI patients are similar to those in the general population	SCI increases the risk for coronary artery disease (CAD) and pulmonary embolus (PE)
Neuro		SCI increases the incidence of peripheral neuropathy. Neuropathic arthropathy of the spine can have imaging features similar to infection. Aspiration and culture may be necessary to distinguish
Bone and soft tissue	Reliable distinction of cellulitis from abscess formation requires the administration of contrast media on CT and MR. Abscess will typically show rim enhancement around a central non-enhancing fluid collection	Lower extremity joint effusions seen in SCI patients may be aseptic or infectious. Spinal hardware radiographs should be carefully compared to prior imaging to detect loosening and failure

*Source: Gunn ML, ed. Pearls and Pitfalls in Emergency Radiology, Cambridge Medicine, 2013

## Genitourinary findings and complications

**Fig. 1:**
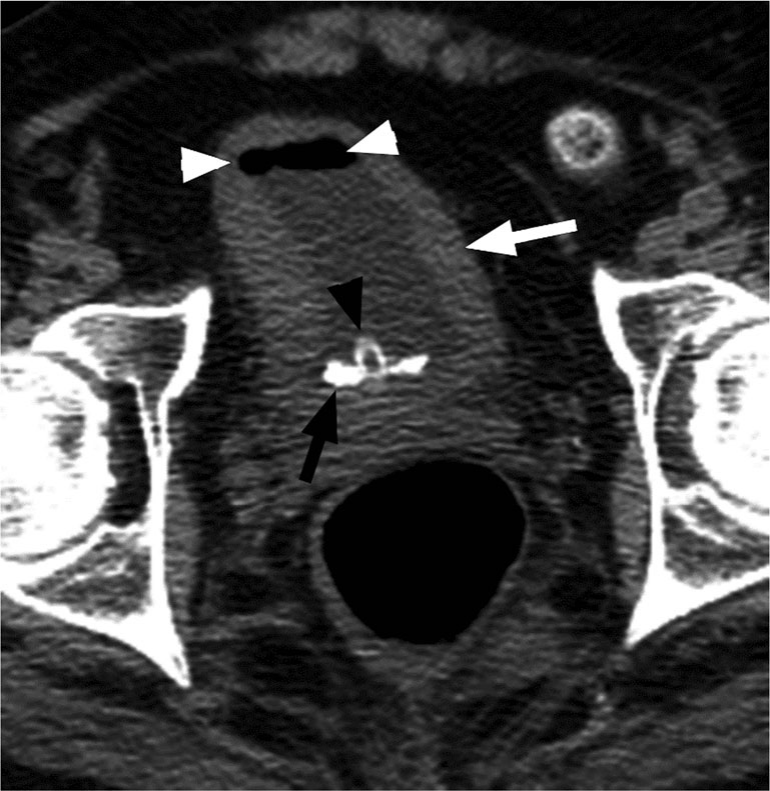
Neurogenic bladder with bladder stone formation. SCI patient with indwelling urinary catheter (*black arrowhead*). Non-contrast CT image shows circumferential urinary bladder wall thickening (*white arrow*) and a calcified bladder stone layering dependently (*black arrow*). Note catheter tubing within the bladder lumen (*black arrowhead*) causing gas in the urinary bladder (*white arrowheads*)

Spinal cord injury can cause bladder dysfunction, often referred to as a neurogenic bladder. Bladder function is complex, with parasympathetic control of bladder contraction and sympathetic and voluntary control of the bladder neck and sphincter. The clinical picture varies based on the injury acuity, level and completeness. Bladder detrusor muscular hyperactivity results in reflexive bladder emptying, spasms, urgency and frequency, often with incontinence. In the chronic setting there is decreased bladder capacity. The most commonly encountered imaging finding is a small, thick-walled urinary bladder (Fig. 1) [6–8]. Trabeculations may be evident on imaging. An unusual contour, described as an hourglass configuration, can result from detrusor hyperactivity, or secondary to a malpositioned suprapubic catheter with excessive traction applied to the bladder dome, but this imaging feature is more apparent on cystography [9]. Sphincter hyperactivity with incomplete emptying and detrusor-sphincter dyssynergia with bladder contractions against a closed sphincter can lead to high bladder pressures, significant post-void residual bladder volumes and vesicoureteral reflux (VUR). Alternatively, there may be decreased detrusor muscle tone with bladder flaccidity, chronic urinary retention, overflow incontinence and incomplete emptying. At imaging, the bladder is enlarged and thin-walled, and VUR or secondary findings to suggest VUR, such as dilated renal collecting systems and ureters with renal cortical scarring, are often present. Imaging findings of neurogenic bladder are often complicated by secondary stone formation and infection [6–8].

**Fig. 2:**
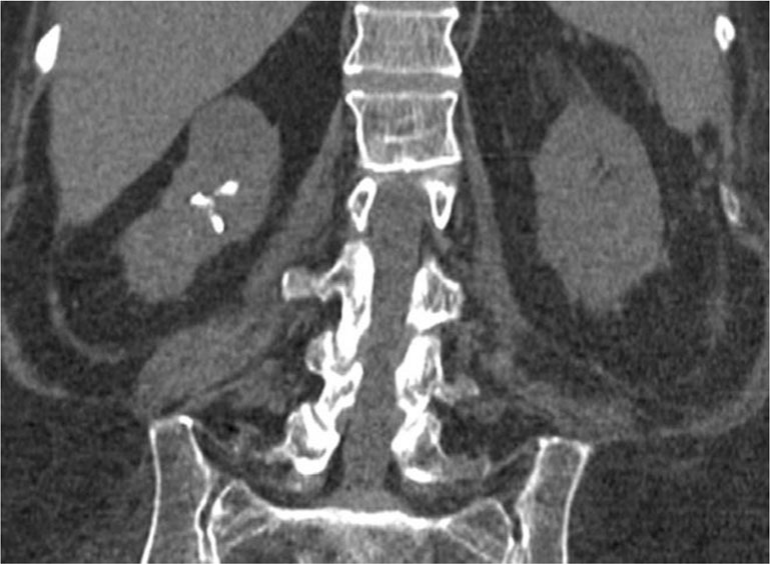
Renal calculi: multiple right renal calculi with mild cortical thinning and parenchymal loss in this patient with remote spinal cord injury. Routine urologic follow-up is an essential component in the care of spinal cord injury patients to prevent the consequences of acute obstructive processes and to preserve renal function

Most patients with SCI require augmented methods for bladder emptying. Clean technique intermittent catheterisation has a lower infection rate compared to indwelling catheters; however, some patients will require an indwelling catheter (Table 1). Catheter-dependent patients are at increased risk of urinary tract infection, prostatitis, epididymitis, orchitis, scrotal/perineal abscess and urethral strictures [10, 11]. There is an increased risk of bladder cancer and stone formation in patients with neurogenic bladders, and this risk is increased further in the setting of chronic catheterisation. Routine genitourinary surveillance imaging has been advocated, although there is no consensus as to the best imaging method or frequency [12]. Most SCI providers evaluate upper and lower tract functioning annually with more frequent urological evaluations if an individual is having problems [13]. In addition to an indwelling catheter, recurrent UTI, vesicoureteral reflux and immobilisation hypercalciuria are important risk factors for the development of urolithiasis in SCI patients [10]. At the authors’ institution, SCI patients are monitored annually by renal ultrasound and annually to biannually with abdominal radiographs to detect asymptomatic stone disease or hydronephrosis, as well as to detect renal parenchymal loss. Figure 1 shows an asymptomatic bladder stone in an SCI patient with a chronic indwelling catheter, and Fig. 2 demonstrates multiple renal calculi and mild cortical thinning in an SCI patient who underwent imaging for another indication.

**Fig. 3:**
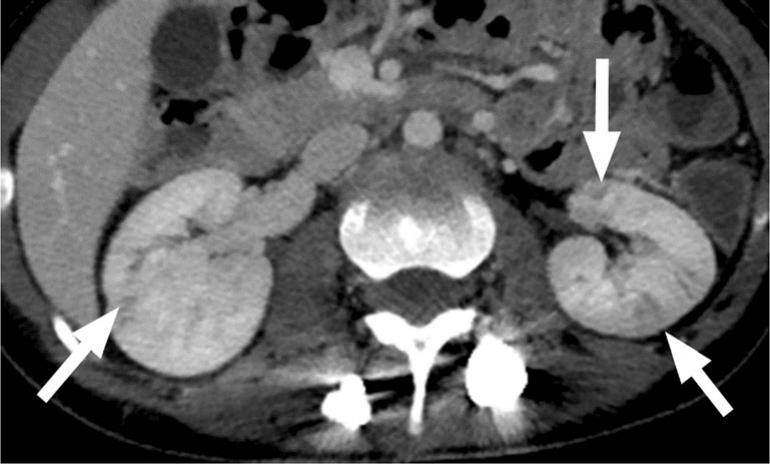
Pyelonephritis: a 26-year-old paraplegic female who presented with diffuse abdominal pain. Axial image from contrast-enhanced CT shows striated enhancement of the kidneys bilaterally, with white arrows indicating linear regions of decreased enhancement

**Fig. 4:**
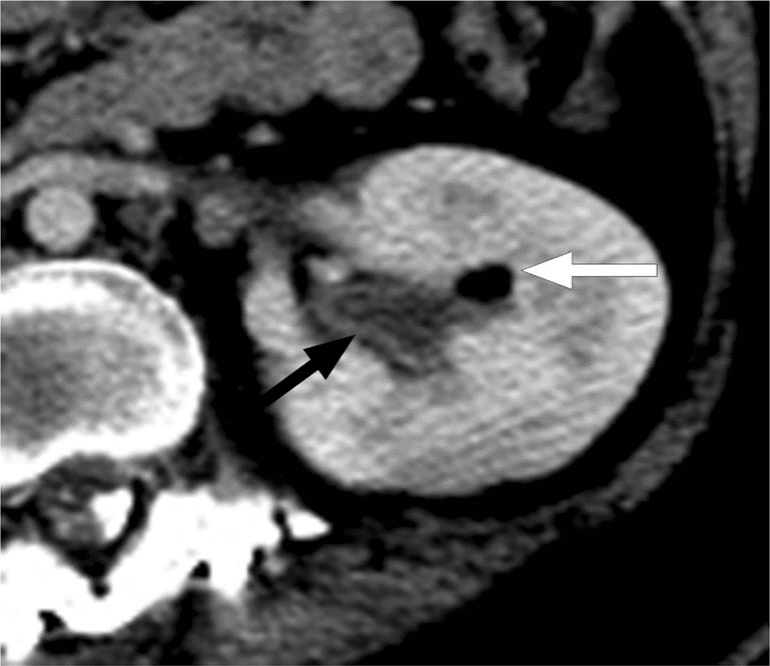
Pyelitis. Axial image from contrast-enhanced CT shows air within the left renal collecting system (*white arrow*). There is uroepithelial thickening and enhancement (*black arrow*)

**Fig. 5:**
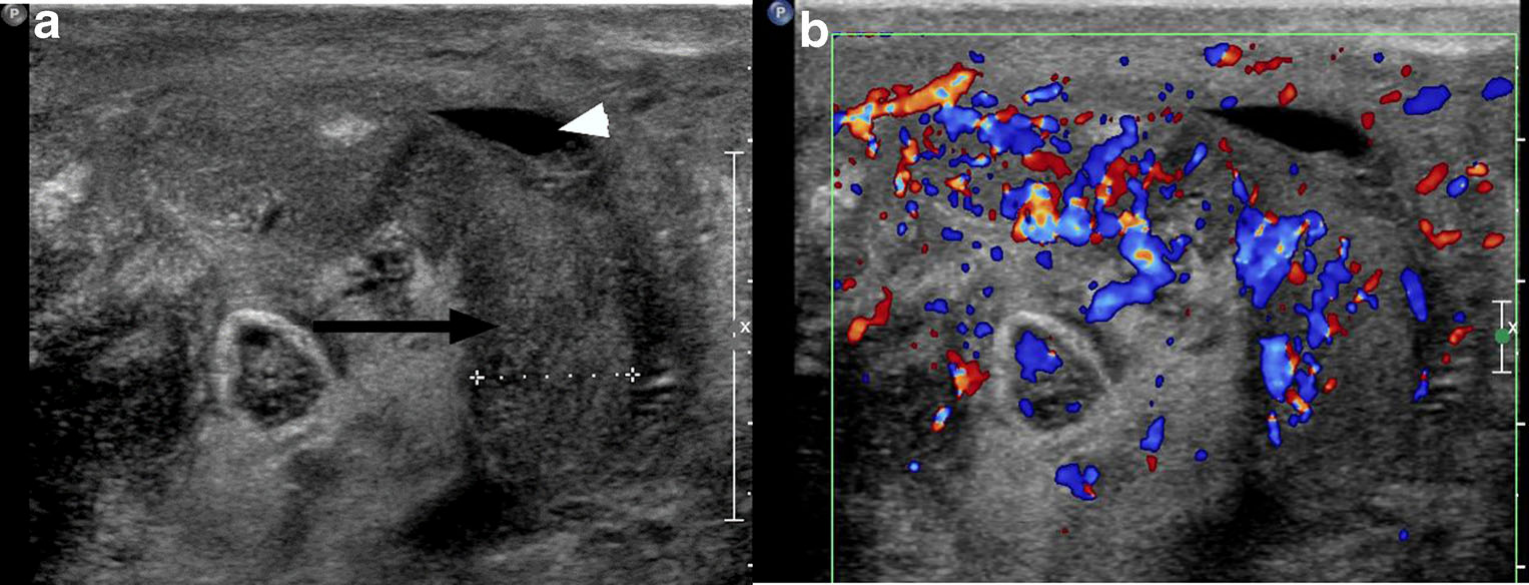
Epididymitis: a paraplegic male who presented to the ED with a painful, high-riding testicle. Image from scrotal ultrasound reveals the enlarged, heterogeneous body of the epididymis (*black arrow*). There is a small associated hydrocele (*white arrowhead*). Color Doppler image of the epididymis confirms hyperaemia

Urinary tract infection (UTI) is common after SCI, with an incidence of 2.5 episodes per patient per year. It is important to recognise that UTI is a frequent source of septicaemia and has a mortality rate of approximately 15 % in the SCI population [14]. Imaging findings are similar to those seen in patients without SCI (Fig. 3). While there is an increased incidence of cystitis and pyelonephritis in persons with SCI, the absence of symptoms or presence of non-specific symptoms such as fever or autonomic dysreflexia, a manifestation of the loss of coordinated autonomic responses to demands on the heart rate and vascular tone, often leads to a delay in diagnosis. At the time of clinical presentation, the patient may have developed complications or widespread infection or sepsis [8, 10, 15, 16]. Figure 4 shows gas within the renal collecting system secondary to infection with a gas-forming organism or pyelitis. This serious infection requires urgent drainage and has a mortality rate approaching 20 %. It is important to distinguish from emphysematous pyelonephritis, in which there is gas in the renal parenchyma, necessitating emergency nephrectomy, and which has a mortality rate of nearly 80 % in this population [15, 17, 18]. Epididymitis is a common complication in SCI, associated with voiding dysfunction and retrograde spread of organisms from the urinary bladder or prostate. At ultrasound, the epididymis is enlarged, hypoechoic and hypervascular (Fig. 5) [15, 17]. The role of imaging in the evaluation of the genitourinary system in SCI patients is to assess for obstruction and to identify an underlying cause such as calculi; to evaluate for signs of active infection; to identify or exclude serious complications, such as renal or perinephric abscess or emphysematous pyelonephritis requiring emergency intervention; and to detect chronic changes such as scarring.

**Fig. 6:**
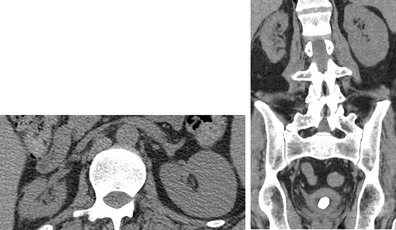
Renal atrophy in an SCI patient secondary to chronic vesicoureteral reflux. The right kidney is small with cortical thinning. Note also the thick-walled urinary bladder containing a large, calcified bladder stone

**Fig. 7:**
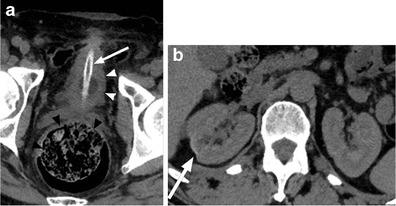
Suprapubic catheter and renal scarring. An SCI patient with an indwelling suprapubic catheter for 15 years. **a** Small bladder volume with wall thickening (*white arrowheads*) and a suprapubic indwelling catheter (*white arrow*). Note the faecal impaction in the rectum (*black arrowheads*). **b** Focal renal cortical scarring of the right kidney (*white arrow*)

Vesicoureteral reflux may result from high bladder pressures and recurrent UTIs. The estimated incidence of reflux in SCI is as high as 25 % [19, 20] (Figs. 6 and 7). End-stage renal disease is a known complication of SCI, resulting from a combination of UTI, calculous disease, obstruction, reflux and/or secondary amyloidosis. Renal insufficiency increases with time since SCI and is as high as 25 % at 20 years post-injury [10, 21, 22].

## Gastrointestinal findings and complications

**Fig. 8:**
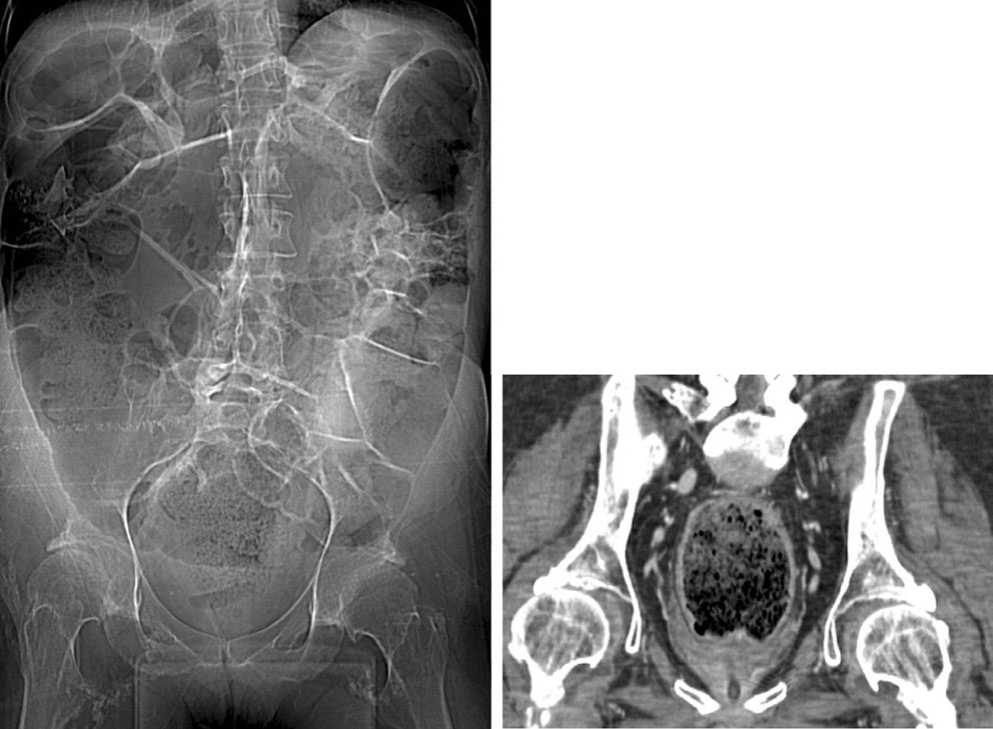
Faecal impaction: scout radiograph of the abdomen demonstrates a large volume of stool throughout the colon and rectum. Coronal CT image confirms large rectal stool ball with mild associated rectal wall thickening, compatible with faecal impaction

Neurogenic bowel, bowel dysfunction resulting from a neurological lesion such as SCI, has a significant effect on quality of life and functional outcomes [23–25]. SCI above the level of the conus results in hyperreflexic pelvic muscular contraction and an inability to relax the external anal sphincter. These patients develop constipation and faecal retention. Spinal cord injury below the conus results in slower gastrointestinal transit and decreased sphincter tone, clinically presenting with constipation and faecal incontinence [26]. In the emergency room setting, SCI patients often present with faecal impaction, which is readily detected by radiograph or CT, often obtained to screen for obstruction (Fig. 8).

There is an increased prevalence of gallstones in SCI patients, as well as an increased mortality associated with complications of cholelithasis due to delayed diagnosis in this population [27]. Non-specific symptoms such as nausea, anorexia or autonomic dysreflexia should prompt evaluation for acute abdominal complications such as cholecystitis or pancreatitis (Table 1). While physicians should have a low threshold to obtain gallbladder imaging, the sonographic and CT findings are straightforward and similar to those for the general population.

**Fig. 9:**
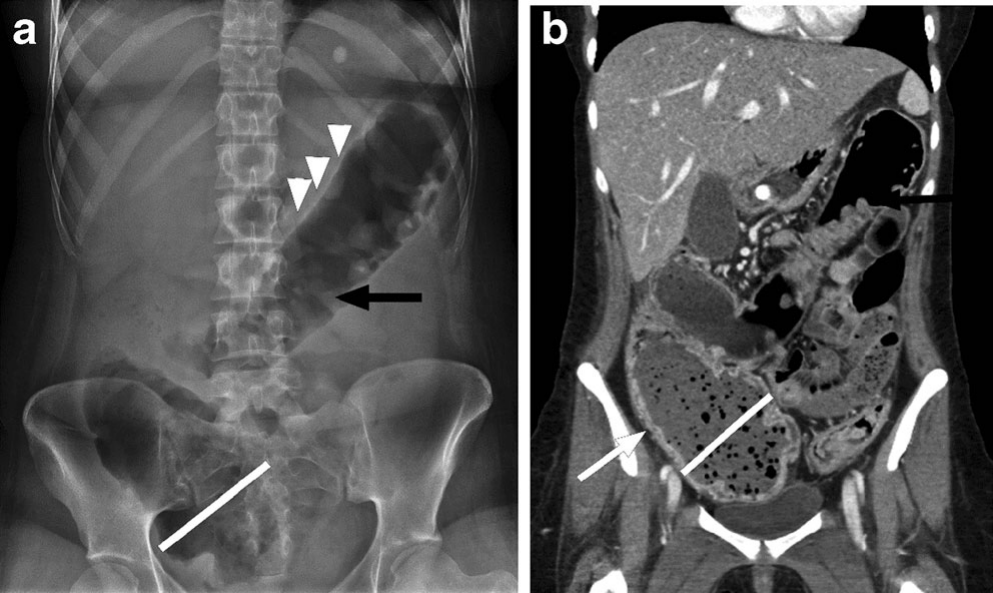
**a** Abdominal radiograph of a 20-year-old female with SCI and abdominal pain shows a dilated ascending and transverse colon (*white line*) with loss of colonic haustrations (*white arrowheads*) and pseudo-poly formation of the colonic mucosa (*black arrow*), concerning for toxic mega colon. **b** The corresponding coronal CT images confirm radiographic findings of bowel dilatation (*white line*), bowel wall thickening and oedema (*white arrow*) and mucosal pseudo-polyps (*black arrow*) typically associated with toxic megacolon

Owing to the presence of sensory impairments, patients with SCI may experience delayed diagnosis in the setting of abdominal pathology. One particularly concerning diagnosis for which patients with SCI may also be at higher risk than the general population is toxic megacolon. This is an important diagnosis to remain aware of because of its potential lethality (Fig. 9).

**Fig. 10:**
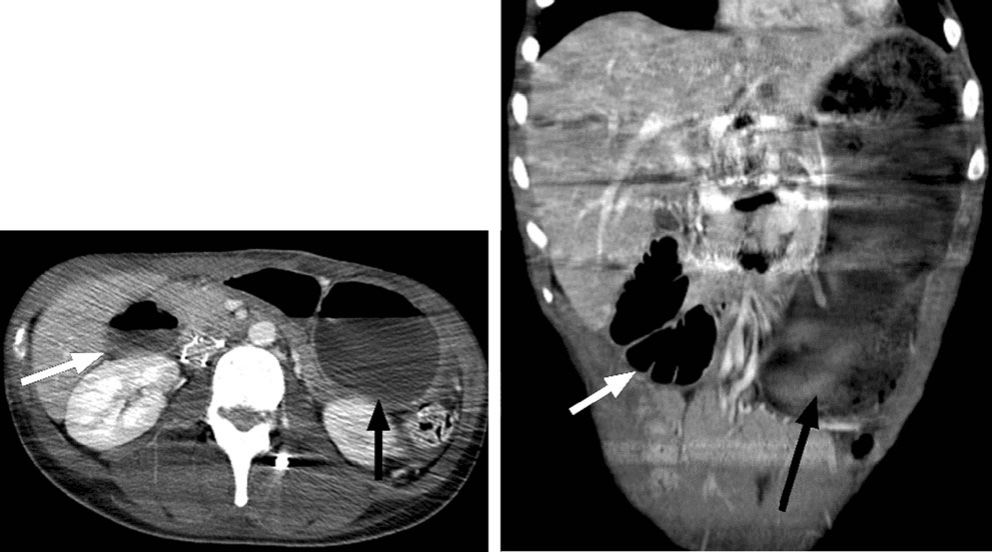
SMA syndrome. Axial and coronal CT images show the distended, fluid-filled stomach (*black arrows*) and mild dilation of the proximal duodenum (*white arrows*). The duodenum is narrowed as it crosses the spine under the superior mesenteric artery and the remaining small bowel is small in caliber (not shown). Note the substantial streak artifact from the spinal fusion hardware

An unusual complication of SCI is superior mesenteric artery syndrome. This occurs more commonly in patients with cervical SCI. Loss of mesenteric fat occurs in the setting of significant or rapid weight loss, allowing the duodenum to be compressed between the superior mesenteric artery and the spine, leading to symptoms of small bowel or gastric obstruction (Fig. 10) [28, 29]. Marked distention of the stomach can occur and, if not appreciated, can progress to ischaemia.

## Cardiovascular and pulmonary findings and complications

Coronary artery disease prevalence is 3–10 times higher in SCI patients compared to the general population [30]. Factors that may contribute to the increased prevalence include changes in body composition that result from inactivity such as decreased muscle mass, increased adiposity and dyslipidaemia. Interestingly, mortality from coronary artery disease is also higher than in the general population, possibly because of the atypical presentation of ischaemia in SCI patients with injuries above T5 [2] (Table 1). Patients with atypical chest pain and low to intermediate risk for coronary artery disease are often evaluated with coronary CTA, which allows safe discharge from the emergency department if negative.

As above, autonomic dysreflexia is a condition resulting from the loss of coordinated autonomic responses to demands on the heart rate and vascular tone. This syndrome is unique to persons with cervical or high thoracic spinal cord injury and is caused by the interruption of sympathetic inhibition that occurs with SCI. Autonomic dysreflexia is triggered by noxious stimuli below the neurological level of injury and presents with severe hypertension as well as possible headache, flushing, nasal congestion and piloerection. This syndrome must be recognised swiftly, as the severe hypertension can have serious sequelae. The symptoms can be managed while the aetiology is being determined, but resolution of the causative noxious stimuli is the ultimate treatment.

**Fig. 11:**
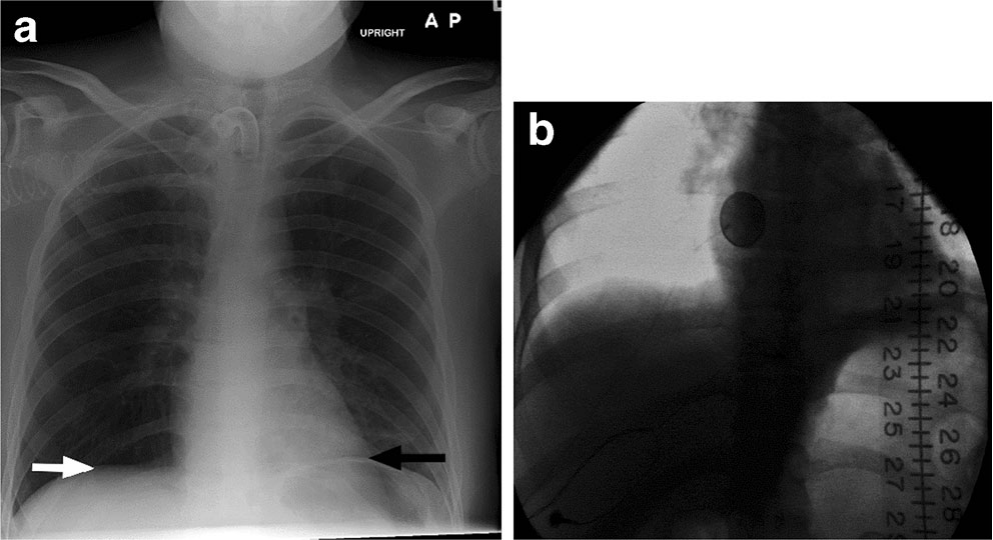
**a** Anteroposterior radiograph of a 29-year-old male with C2 tetraplegia from a diving accident shows elevation of the left hemidiaphragm (*black arrow*) in relation to the right (*white arrow*), concerning for diaphragmatic paralysis. **b** Fluoroscopic spot image from the subsequently obtained “sniff” test confirms the diagnosis

Impaired pulmonary function, ineffective cough and difficulty mobilising secretions place SCI patients at increased risk for atelectasis and aspiration. There is also an increased risk of pneumonia throughout their lifetime, although the risk is greatest in the first year following injury [10]. Patients with SCI are also at risk for tracheal stenosis after tracheostomy. High cervical spinal cord injury can cause diaphragmatic paralysis. This diagnosis may be noted on chest radiographs and can be confirmed using fluoroscopy to perform a “sniff” test (Fig. 11).

Deep venous thrombosis (DVT) and pulmonary emboli (PEs) are common early complications of SCI despite advances in awareness and treatment. Prophylaxis with low-molecular-weight heparin is the standard of care for patients with new SCIs for 8–12 weeks post-injury depending on risk factor stratification [31]. After 3 months, the risk of DVT and PE approaches that of the general population [32].

## Neurological findings and complications

**Fig. 12:**
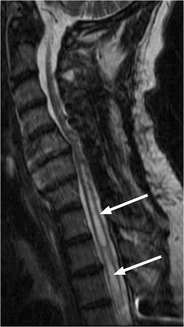
Syrinx: T2-weighted sagittal image from cervical spine MRI that shows expansion of the spinal cord with central cyst formation extending from the C6 level into the upper thoracic spine (*white arrows*)

Chronic changes identified in the spinal cord following injury include atrophy, myelomalacia, syrinx, focal cyst formation and cord disruption. Delayed progressive intramedullary cystic degeneration complicates 3–4 % of traumatic SCIs (Fig. 12). This progression to syringomyelia is felt to involve arachnoid adhesions, presumably secondary to haemorrhage and inflammation at the time of injury, which alters and/or obstructs the flow of cerebral spinal fluid and leads to expansion of the central canal [33]. Risk factors for progression of syringomyelia and neurologic deterioration include arachnoiditis, cord compression and kyphosis [33, 34]. Treatment is reserved for symptomatic or expanding syrinx with the goal to restore CSF flow dynamics.

**Fig. 13:**
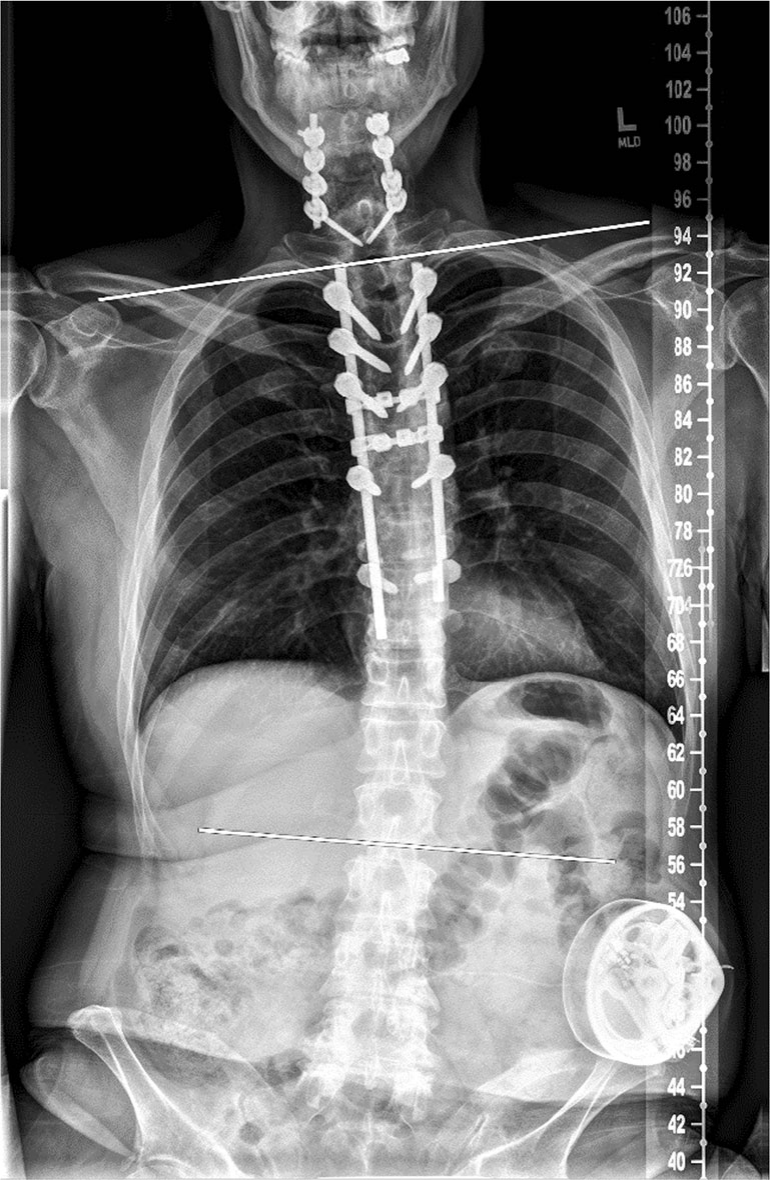
Neuromuscular scoliosis: upright scoliosis radiograph of a 51-year-old male (T6 SCI from a snowboarding injury 18 months earlier) shows neuromuscular dextroscoliosis. The scoliosis angle is 18° measured between the endplates of T2 and L2 (*white lines*). Anteroposterior alignment was normal (not shown)

Neuromuscular scoliosis is a known complication of SCI. The patient age at time of injury is a major determinate in the severity of spinal deformity. Ambulatory status is the other major determinate, with more severe deformity in the non-ambulatory patients. Progressive curvature causes difficulty with transfers and sitting upright (Fig. 13). In contrast to idiopathic juvenile scoliosis, bracing is ineffective in the treatment of neuromuscular scoliosis as patients typically do not possess the motor function to contract the axial muscles to help correct their curvature. Surgical correction may be required for severe or progressive cases. Laminectomies in the thoracic spine at the time of injury can result in progressive post-laminectomy kyphosis [35, 36].

The incidence of peripheral mononeuropathies increases with the number of years following SCI, with nearly two-thirds of spinal cord-injured patients developing upper extremity compressive neuropathies (Table 1). Median neuropathies account for over 50 % of cases, with a significant number bilaterally. These overuse injuries, in particular, carpal tunnel syndrome, are a frequent cause of hand pain. Even in the absence of clinical symptoms, electrophysiologic abnormalities of the median nerve are present in 26 % of SCI patients compared to approximately 6 % of asymptomatic controls [37–39].

**Fig. 14:**
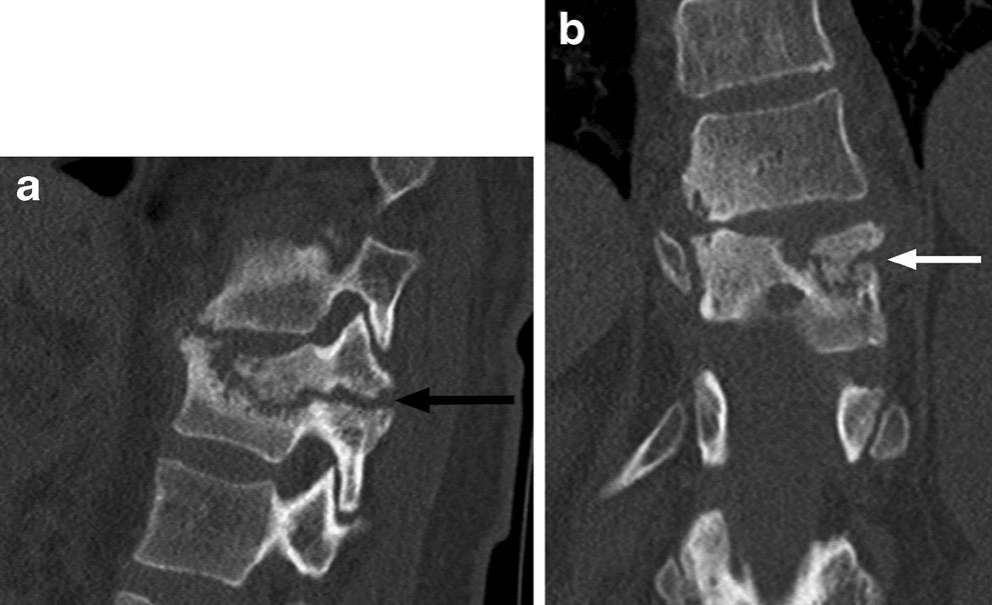
Charcot spine in a 32-year-old female with SCI due to a motor vehicle crash 2 years prior. **a** Parasagittal CT image of the left T11 pedicle shows development of an irregular pseudarthrosis with sclerosis and fragmentation (*black arrow*). **b** Coronal CT image depicts the pseudarthrosis (*white arrow*) and disc space narrowing above it. There is associated mild dextroscoliosis

Neuropathic arthropathy of the spine, or Charcot spine, is relatively uncommon. This degeneration of the spine occurs as a progressive cycle of instability and destruction in persons with diminished or absent sensation. Clinical symptoms include pain, loss of spasticity, change in bowel or bladder function, loss of sitting balance and skin ulceration [40]. Imaging findings include vertebral body height loss, disc degeneration, endplate resorption and progression to gross subluxation and destruction (Fig. 14). Neuropathic changes and osteomyelitis can have similar imaging features and can co-exist. Diagnosis may require aspiration/biopsy and culture. Treatment is difficult as there is a high rate of surgical failure and post-surgical complications such as deep infections, need for repeat surgeries and the development of new Charcot levels [40].

## Osseous and soft tissue findings and complications

**Fig. 15:**
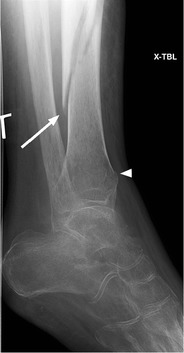
Marked osteopaenia and spiral tibial shaft (*white arrow*) and metaphyseal (*white arrowhead*) fractures in a patient with a remote SCI. These patients are at high risk for limb fractures, often occurring with only minor trauma, such as bed or wheelchair transfers

**Fig. 16:**
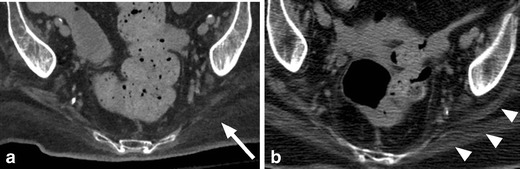
Marked muscular atrophy in patient 7 years after SCI. **a** Non-contrast CT image shows marked atrophy of the gluteal musculature with fatty replacement (*white arrow*). **b** The same patient, 7 years earlier, demonstrating baseline muscle mass (*white arrowheads*)

Following SCI, osteoporosis affects the bones below the level of injury, which manifests clinically with an increased incidence of lower extremity fractures following only minor trauma (Fig. 15) [41–44]. Extensive loss of muscle mass below the level of injury is commonly seen and an expected imaging finding in SCI patients due to muscle disuse and atrophy (Fig. 16) [17].

**Fig. 17:**
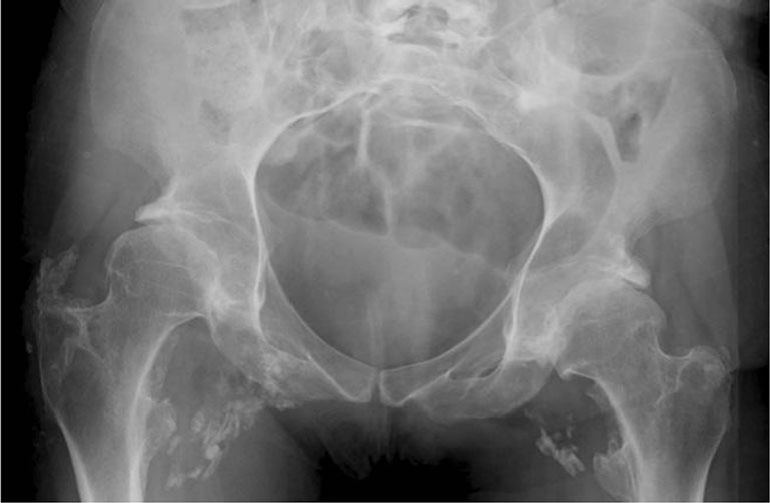
Heterotopic ossification: a patient 2 years after SCI, with soft tissue ossification involving both hips. The hip is the most commonly involved joint

**Fig. 18:**
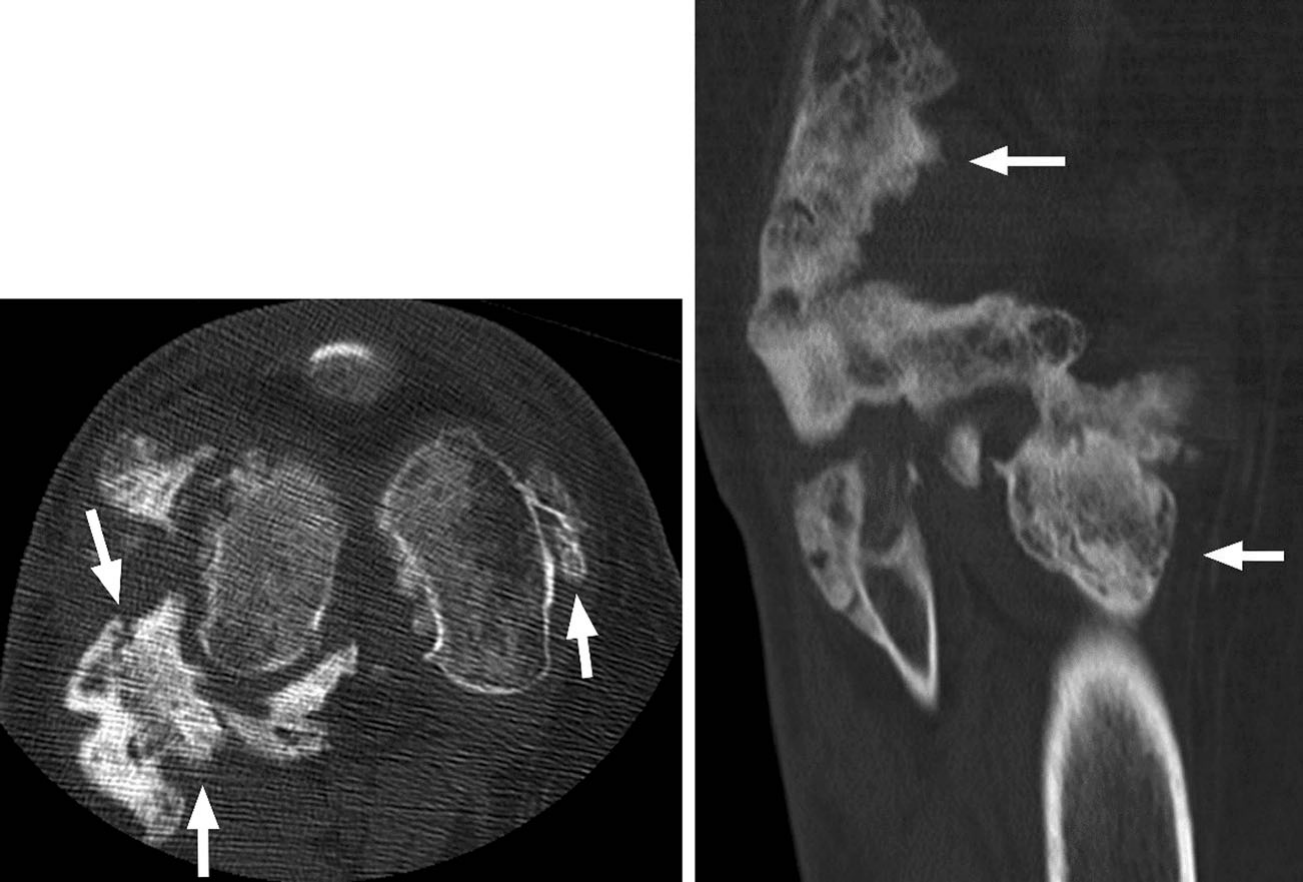
Heterotopic ossification: large soft tissue ossification bridging the knee joint (*white arrows*). This patient presented with chronic lower extremity swelling and decreased range of motion

**Fig. 19:**
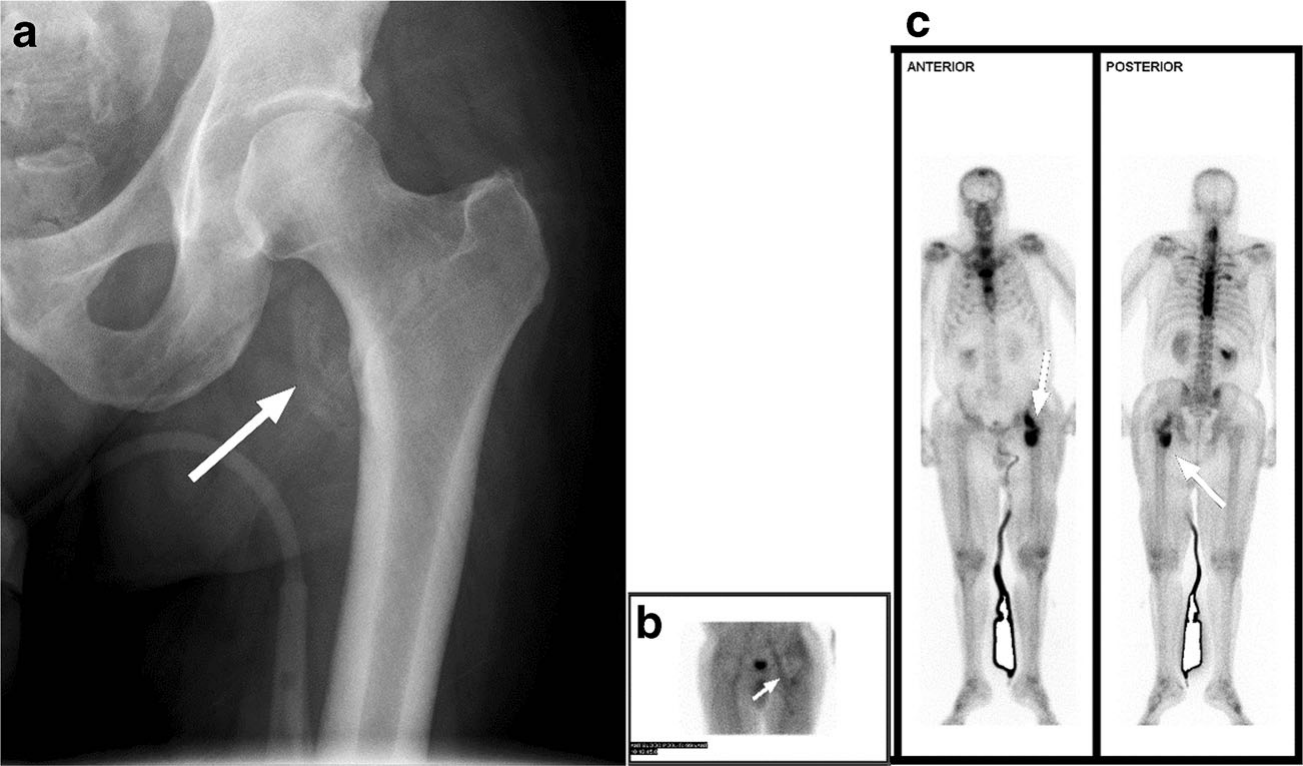
Heterotopic ossification. **a** Hip radiograph of a 46-year-old male 3 months after T4 SCI shows ill-defined calcifications medial to the lesser trochanter (*white arrow*). **b** Blood pool image of the subsequently obtained triple-phase bone scintigraphy shows hyperaemia in the left groin (*white arrow*). **c** Delayed bone scintigraphy images confirm avid tracer uptake medial to the left proximal femur (*white arrows*). Bone scan findings confirm maturing heterotopic ossification

Heterotopic ossification (HO), or abnormal periarticular bone formation within the soft tissues around the peripheral joints, occurs in approximately half of patients with SCI. On average, the process begins 12 weeks following injury and is probably related to repetitive microtrauma [8, 45]. However, only 10–20 % will experience symptoms such as a decreased range of motion or inflammation of the involved joint. This process most commonly affects the large joints of the lower extremity, with the hip involved more often than the knee (Figs. 17 and 18) [46, 47]. Patients may present with an oedematous leg and HO must be differentiated from deep venous thrombosis or cellulitis. Conventional radiographs are of limited use, as radiographic findings may not be apparent for several weeks following presentation. CT is sensitive for the detection of HO; however, a triple-phase bone scan is the most reliable test for diagnosis early in the disease process (Fig. 19). Bone scintigraphy is also useful to monitor activity prior to surgical excision [8].

**Fig. 20:**
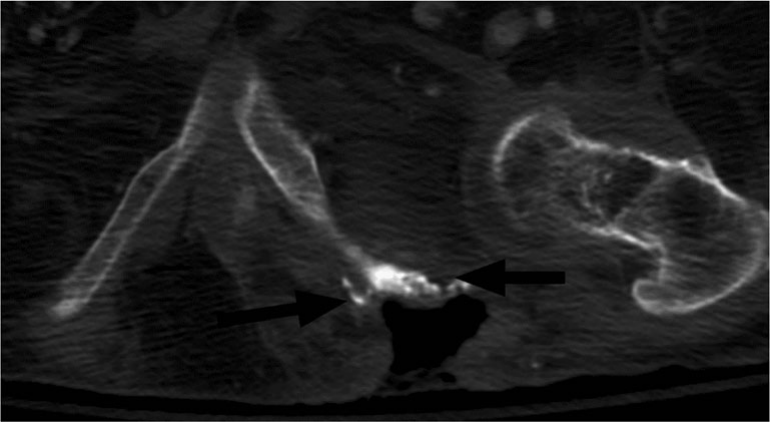
Decubitus ulcer and chronic osteomyelitis. Large skin and soft tissue defect extending to the left ischium, which is irregular, with osseous remodelling and sclerosis (*black arrows*). A left hip effusion is present as well

**Fig. 21:**
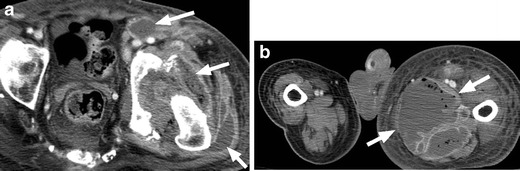
Septic hip effusion with extensive soft tissue infection and abscess. **a** Fluid and gas are present in the left hip joint. The joint space is widened and there is osseous destruction and fragmentation along the margins of the acetabulum. Numerous rim-enhancing abscesses are present surrounding the hip and within the soft tissues of the proximal left thigh, (*white arrows*). **b** Marked asymmetry of the lower extremities with enlargement of the left leg and a large soft tissue abscess in the medial left thigh (*white arrows*)

Due to the mobility impairments, sensory deficits and skin changes associated with SCI, SCI patients are extremely susceptible to pressure sores. Once developed, secondary infection is common and septic arthritis and osteomyelitis can result. Decubitus ulcers are commonly encountered at imaging and it is paramount to carefully assess the extent and depth of the lesion [8, 10]. While bone scintigraphy reliably allows the distinction of cellulitis from osteomyelitis, it is rarely used as the first line imaging modality in the emergency setting owing to the unavailability of radioisotopes at night, prolonged scan time and poor soft tissue delineation. Figure 20 shows a large soft tissue defect extending to the surface of the left ischium with osseous changes typical of chronic infection. Osseous changes concerning for infection include periosteal reaction, sclerosis, focal cortical erosion and frank bone destruction, often encountered on CT and radiography. However, MRI is useful to evaluate for osteomyelitis, determine the extent of involvement and assess for an associated deep soft tissue abscess, especially if uncertainty regarding osseous involvement exists [48]. In our practice, MRI is generally used as a problem-solving tool. Figure 21 illustrates wide-spread soft tissue infection and abscess formation with septic hip joints (Table 1).

**Fig. 22:**
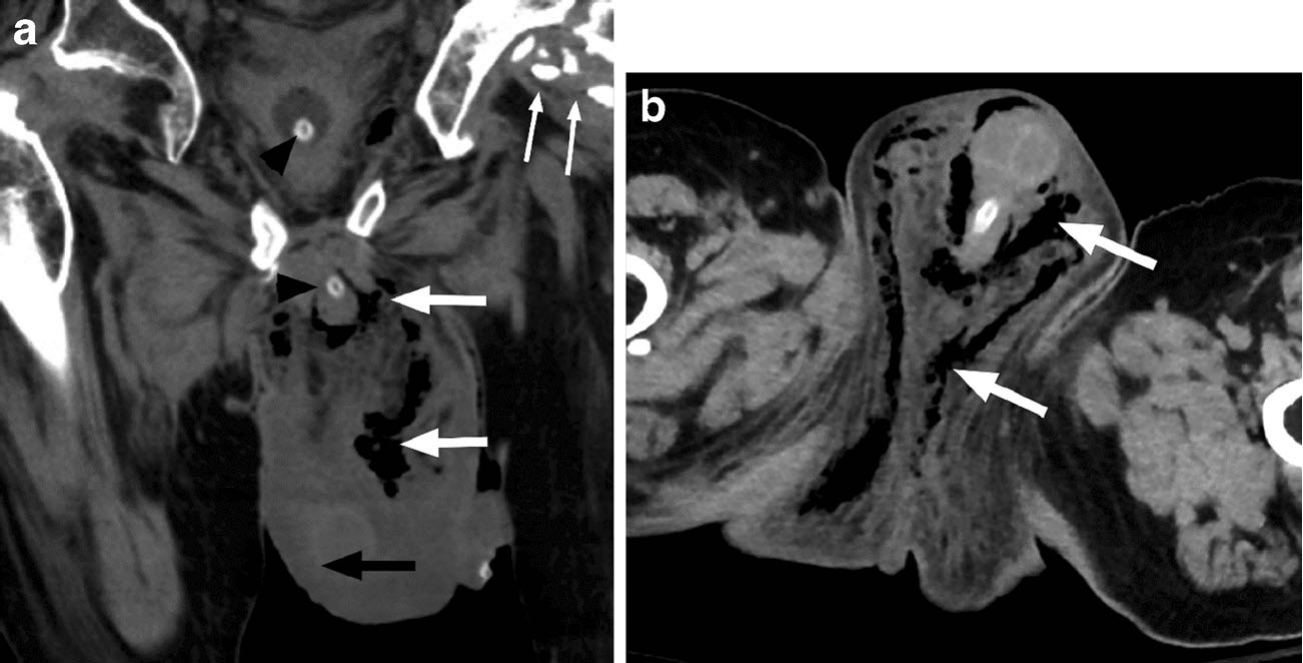
Fournier’s gangrene. **a** Coronal non-contrast CT image shows scrotal wall oedema (*black arrow*), with extensive subcutaneous gas in the scrotum and perineum (*white arrows*). Also note the presence of a bladder catheter (*black arrowheads*) and heterotopic ossification around the left hip (*small white arrows*). **b** Axial image from the same patient demonstrating the extent of infection and presence of gas throughout the perineum and scrotum (*white arrows*)

Fournier’s gangrene is a potentially life-threatening necrotising fasciitis of the perineum and genital region requiring rapid diagnosis and treatment (Fig. 22). Initial symptoms are severe pain in the genital region followed by swelling and erythaema. In patients with SCI, lack of pain sensation often delays diagnosis. Risk factors for the development of necrotising perineal fasciitis include immunosuppression, paralysis, skin ulceration, peripheral vascular disease, renal insufficiency and instrumentation or catherisation. Adjunct CT imaging may be useful to confirm diagnosis, define the extent of disease and plan surgical treatment. Treatment requires early, aggressive antibiotics and surgical debridement, which should not be delayed by imaging [48, 49].

**Fig. 23:**
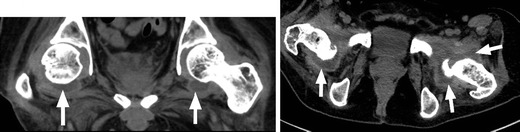
Benign hip effusions. Large bilateral hip effusions (*white arrows*) detected incidentally on CT abdomen and pelvis obtained for another indication. These were found to be sterile effusions in this patient without fever or elevated white blood cell count

Benign, sterile lower extremity joint effusions can be encountered in paralysed patients (Fig. 23). The aetiology is not well understood. It has been hypothesised that paralysed muscles result in joint laxity thereby allowing for joint fluid accumulation [50]. Others contend that repetitive microtrauma, such as encountered in aggressive range of motion exercises, may be the aetiology [51, 52]. However, SCI patients may develop joint effusions of any cause, infectious, inflammatory, traumatic or benign. It is important to consider the clinical context and exclude a septic joint prior to arriving at the diagnosis of a benign effusion. When there is uncertainty, joint aspiration should be performed.

**Fig. 24:**
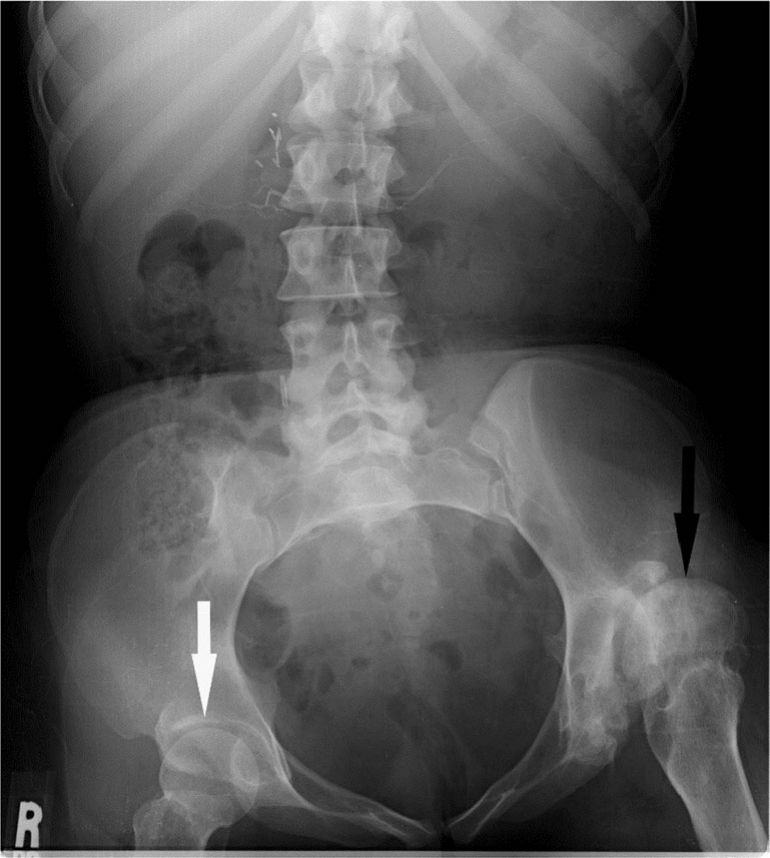
Abdominal radiograph of a 33-year-old female with thoracic SCI from a motor vehicle collision 15 years prior shows superior subluxation of the left femoral head (*black arrow*) due to spasticity. The right femur (*white arrow*) is normally aligned

**Fig. 25:**
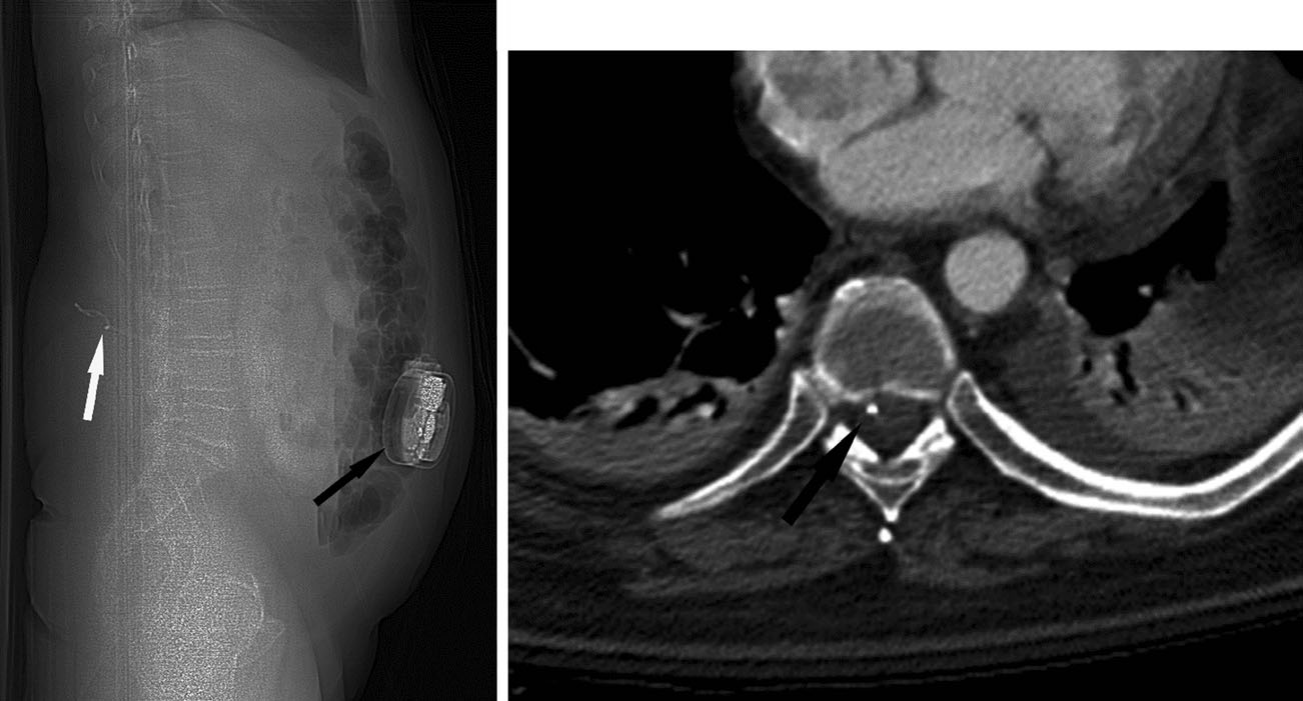
**a** The lateral scout image from an abdominal CT of a 39-year-old male with C2 SCI from a fall 4 years prior shows an intrathecal baclofen pump in the right lower abdominal quadrant (*black arrow*). The tubing from the pump extends posteriorly (*white arrow*) before entering the thecal canal. **b** Axial CT image of the same patient confirms the intrathecal position of the catheter tip (*black arrow*)

Depending on the neurological level of injury and completeness, SCI causes variable patterns of muscle spasticity and weakness. Both weakness and spasticity may result in joint subluxation and dislocation (Fig. 24). Spasticity is a complication of central nervous system damage that can be severe and very disabling. Although less invasive management strategies are often employed, patients with SCI not uncommonly require an implantable intrathecal baclofen pump to adequately manage their spasticity. Due to the possibility of pump or component malfunction or infection, it is important to be familiar with imaging of these pumps (Fig. 25).

**Fig. 26:**
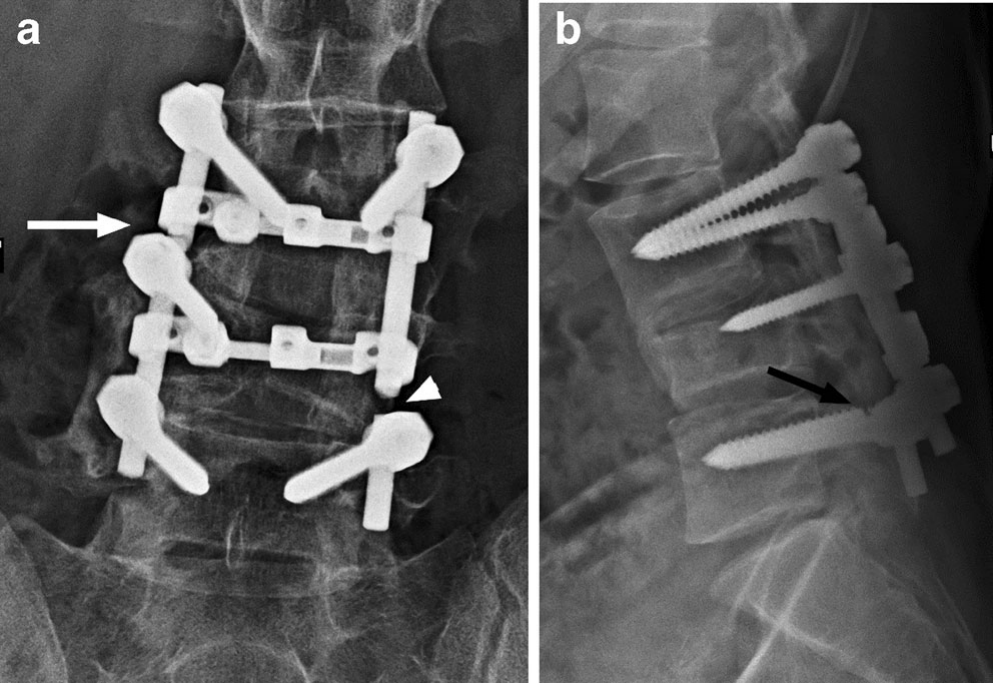
Hardware complication: a 24-year-old male presents with back pain 24 months after posterior segmental instrumentation and fusion of L3 to L5 for SCI because of a crush injury. **a** Antero-posterior radiograph shows disruption and distraction of the left fusion rod (*white arrowhead*). Failure of the right fusion rod is less conspicuous (*white arrow*). **b** The lateral radiograph demonstrates a break in the L5 pedicle screw (*black arrow*). Follow-up radiographs have to be carefully scrutinised for the presence of hardware failure and comparison with priors is essential

Hardware complications and infection can prove a diagnostic challenge in patients with SCI as classic symptoms such as pain may be absent. Spinal hardware should be carefully assessed on all imaging studies to exclude hardware fracture or loosening (Fig. 26). Vertebral osteomyelitis should be considered in febrile SCI patients, even in a patient with a known site of infection [53].

## Conclusion

SCI patients present a unique diagnostic challenge as they may present with severe infection that is difficult to localise because of abnormal sensation and autonomic instability. Imaging plays an important role in the emergent setting, rapidly differentiating the most commonly encountered complications such as urinary tract infections or obstructions, pressure sores and deep soft tissue infections, and orthopaedic sepsis, as well as identifying less common, unanticipated complications.

When the aetiology of sepsis or suspected sepsis remains occult, one should consider obtaining chest-abdomen-pelvis CT to try to determine the source of infection. In a study of SCI patients (*n* = 22) with suspected or known sepsis, investigators found that chest-abdomen-pelvis CT identified a specific radiologic diagnosis in 14 % and non-specific findings in 68 %. Although the bulk of findings were non-specific, a small portion of patients had significant radiologic findings that influenced their management [54].

Radiologists need to be attuned to both the expected findings and potential complications, which may be unique to SCI patients, to ensure accurate diagnosis and treatment in the emergency setting.
